# Prediction of pre-eclampsia in nulliparous women using routinely collected maternal characteristics: a model development and validation study

**DOI:** 10.1186/s12884-019-2712-x

**Published:** 2020-01-06

**Authors:** Ziad T. A. Al-Rubaie, H. Malcolm Hudson, Gregory Jenkins, Imad Mahmoud, Joel G. Ray, Lisa M. Askie, Sarah J. Lord

**Affiliations:** 1 School of Medicine, The University of Notre Dame Australia, 160 Oxford Street, Darlinghurst, NSW 2010 Australia; 2 NHMRC Clinical Trials Centre, Sydney Medical School, University of Sydney, Level 6 Medical Foundation Building, 92 Parramatta Road, Locked Bag 77, Camperdown, NSW 2050 Australia; 3 Department of Statistics, Macquarie University, Level 6 Medical Foundation Building, 92 Parramatta Road, Camperdown, NSW 2050 Australia; 4 Department of Obstetrics, Westmead Hospital, Suite 110, 9 Norbrik Drive, Bella Vista, Westmead, NSW 2153 Australia; 5 Department of Obstetrics, Auburn and Mount-Druitt and Blacktown Hospitals, Suite 108, 9 Norbrik Drive, Bella Vista, NSW 2153 Australia; 6 Departments of Medicine, Health Policy Management and Evaluation, and Obstetrics and Gynecology, St. Michael’s Hospital, University of Toronto, 30 Bond Street, Toronto, Ontario M5B 1W8 Canada

**Keywords:** Antenatal care, Australia, Maternal health, National Institute of health and care excellence, Pre-eclampsia, Prediction, Risk assessment, Risk prediction model

## Abstract

**Background:**

Guidelines recommend identifying in early pregnancy women at elevated risk of pre-eclampsia. The aim of this study was to develop and validate a pre-eclampsia risk prediction model for nulliparous women attending routine antenatal care “the Western Sydney (WS) model”; and to compare its performance with the National Institute of Health and Care Excellence (NICE) risk factor-list approach for classifying women as high-risk.

**Methods:**

This retrospective cohort study included all nulliparous women who gave birth in three public hospitals in the Western-Sydney-Local-Health-District, Australia 2011–2014. Using births from 2011 to 2012, multivariable logistic regression incorporated established maternal risk factors to develop and internally validate the WS model. The WS model was then externally validated using births from 2013 to 2014, assessing its discrimination and calibration. We fitted the final WS model for all births from 2011 to 2014, and compared its accuracy in predicting pre-eclampsia with the NICE approach.

**Results:**

Among 12,395 births to nulliparous women in 2011–2014, there were 293 (2.4%) pre-eclampsia events. The WS model included: maternal age, body mass index, ethnicity, multiple pregnancy, family history of pre-eclampsia, autoimmune disease, chronic hypertension and chronic renal disease. In the validation sample (6201 births), the model c-statistic was 0.70 (95% confidence interval 0.65–0.75). The observed:expected ratio for pre-eclampsia was 0.91, with a Hosmer-Lemeshow goodness-of-fit test *p-value* of 0.20. In the entire study sample of 12,395 births, 374 (3.0%) women had a WS model-estimated pre-eclampsia risk ≥8%, the pre-specified risk-threshold for considering aspirin prophylaxis. Of these, 54 (14.4%) developed pre-eclampsia (sensitivity 18% (14–23), specificity 97% (97–98)). Using the NICE approach, 1173 (9.5%) women were classified as high-risk, of which 107 (9.1%) developed pre-eclampsia (sensitivity 37% (31–42), specificity 91% (91–92)). The final model showed similar accuracy to the NICE approach when using lower risk-threshold of ≥4% to classify women as high-risk for pre-eclampsia.

**Conclusion:**

The WS risk model that combines readily-available maternal characteristics achieved modest performance for prediction of pre-eclampsia in nulliparous women. The model did not outperform the NICE approach, but has the advantage of providing individualised absolute risk estimates, to assist with counselling, inform decisions for further testing, and consideration of aspirin prophylaxis.

## Introduction

Antenatal guidelines recommend routine risk assessment for pre-eclampsia in early pregnancy and low dose aspirin prophylaxis for women at elevated risk [1–4]. However, approaches for using established maternal risk factors to classify a woman’s risk of pre-eclampsia vary; and Australian guidelines do not offer an explicit approach [1, 2].

The National Institute of Health and Care Excellence (NICE) in the United Kingdom lists moderate and high-risk factors for pre-eclampsia and recommends prophylaxis for women with one or more high-risk factors, or two or more moderate-risk factors [3]. The listed risk factors are readily available maternal characteristics, so this approach has the practical advantage of being widely accessible, including in low-resource settings. However, its predictive performance has not yet been adequately validated in Australian women. Internationally, validation studies of the NICE approach have reported poor to moderate predictive performance [5–11]. One Australian validation study has reported good sensitivity for prediction of pre-eclampsia requiring delivery before 37 weeks, but was limited by a small sample size (*n* = 543, sensitivity 75% (95% confidence interval (CI) 35–97%), false positive rate 22%) [12].

A limitation of risk factor list approaches such as the NICE approach is that common risk factors such as older maternal age and higher body mass index (BMI) are dichotomised as ‘present’ or ‘absent’ ignoring any relationship between the level of these factors and pre-eclampsia risk. A risk prediction model that combines maternal risk factors, and includes all informative numerical values, has the potential advantage of providing an individualised estimate of pre-eclampsia risk that can assist patient counselling and inform clinical management decisions, rather than limiting categorisation to high-risk versus not high-risk. Although there has been a major research effort to develop risk prediction models for pre-eclampsia in early pregnancy, this research has largely focused on the use of specialised tests such as uterine artery Doppler and serum biomarkers [13] which are not available in low-resource settings, nor routinely used in all public antenatal care clinics in Australia.

Globally, pre-eclampsia is reported in 1–8% of pregnancies [14–17]. In Australia, the average rate of pre-eclampsia was 3.3% of mothers who gave birth between 2000 and 2008, declining from 4.6% in 2000 to 2.3% in 2008 [18]. Internationally, nulliparity is classified as a moderate-risk factor [3, 4]. However, in a recent Australian study, we observed the incidence of pre-eclampsia was lower among nulliparous women than all women (parous and nulliparous) (2.5% vs 3.5% respectively) [19].

A woman’s prior pregnancy history of pre-eclampsia is one of the strongest risk factors for pre-eclampsia [4]. More challenging, is identifying women at high-risk of pre-eclampsia in their first pregnancy. Accurate identification and appropriate management of this group has the potential to provide clinical benefits for current and subsequent pregnancies. However, few studies of pre-eclampsia risk assessment tools have targeted this group.

The aims of this study are to develop and validate a pre-eclampsia risk prediction model for nulliparous women that can be used at the first antenatal visit using routinely collected maternal characteristics; and compare its performance with the NICE approach to inform the development of an Australian strategy for pre-eclampsia risk assessment and prevention.

## Methods

### Study design, setting and data source

We conducted a multi-hospital retrospective cohort study of nulliparous women giving birth between 1 January 2011 and 31 December 2014 at three public hospitals (Auburn, Blacktown/Mount-Druitt and Westmead) in the Western Sydney Local Health District (WSLHD). We included all nulliparous women with no previous pregnancies in the study sample. Study data were extracted from the ObstetriX database held by the hospital maternity units. The ObstetriX database collects information for all women attending their first antenatal visit to the discharge of mothers and their babies from the hospital [20].

We excluded women with missing information for pre-eclampsia, parity or candidate risk factors for pre-eclampsia. We also excluded women who were prescribed antiplatelet therapy in the first trimester given the effectiveness of these agents for preventing pre-eclampsia [21].

The primary outcome was the development of pre-eclampsia of any severity or timing. During the study period, pre-eclampsia was defined as hypertension with new onset of significant proteinuria ≥20 weeks’ gestation [22]. Secondary outcomes were early-onset pre-eclampsia (requiring delivery < 34 weeks’ gestation) and preterm pre-eclampsia (requiring delivery < 37 weeks’ gestation).

### Study data

We extracted maternal socio-demographic characteristics (age, country-of-birth, primary language spoken at home and socioeconomic status classified from postcode using the Index of Relative Socio-economic Advantage and Disadvantage from the Socio-Economic Indexes for Areas (SEIFA)) [23], risk factors for pre-eclampsia (listed below); and study outcomes from the ObstetriX database.

For development of the Western Sydney (WS) risk model, we selected 12 candidate risk factors: maternal age, body mass index (BMI), autoimmune disease, chronic hypertension, chronic renal disease, diabetes mellitus (type 1 or 2), multiple (multi-fetal) pregnancy, family history of pre-eclampsia, conception method, ethnicity, socio-economic status, and smoking status. These candidate risk factors were identified from the antenatal guidelines [1–4]; with the addition of conception methods and smoking status which were identified from a systematic review of published risk models [13]. We categorised ethnicity into two groups based on country of birth and primary language spoken at home (Australian/New Zealand-born English speakers; immigrants and non-English speakers). We categorised socioeconomic status into two groups using the SEIFA index (most disadvantaged SEIFA 1–2; most advantaged SEIFA 3–5).

For validation of NICE approach, we classified women with ≥1 high-risk factors or ≥ 2 moderate-risk factors as meeting the criteria of high-risk for aspirin prophylaxis [3], and refer to this group herein as “screen-positive”. All NICE-listed risk factors relevant for nulliparous women are collected in the ObstetriX database.

Women with missing values for study variables were excluded from the analysis requiring that variable.

### Statistical analysis

We assessed the distribution of risk factors measured as continuous variables (age, BMI) visually by plotting a probability distribution curve. We performed a descriptive analysis of maternal risk factors by assessing the frequency of categorical variables as a percentage in all women, then separately for women who developed pre-eclampsia and those that did not.

#### Model development and validation

We split the study sample into two groups for model development and temporal validation by year of infant birth (model development sample 2011–2012, validation sample 2013–2014). For model development, we used a two-stage approach. First, to optimize prediction of pre-eclampsia from age, BMI and other NICE-listed moderate-risk factors we developed a WS ‘base’ model by excluding women with NICE-listed high-risk factors (autoimmune disease, chronic hypertension, chronic renal disease and diabetes (type 1 or 2)). The approach optimizes the model for use for the large majority of women who do not have high-risk factors; and would be sufficient in settings where women with high-risk factors are already referred for further testing and management. Second, we developed a WS ‘full’ model for use in all women, by introducing women with high-risk factors into the development sample, retaining the base model risk score, and estimating coefficients for the high-risk factors. We internally validated the model in the development sample then externally validated it in the validation sample to assess the potential for model overfitting. If the model fit was satisfactory, we planned to refit the model predictors in the entire study sample to develop a WS ‘final’ model. Further details of these analyses are given below.

#### WS base model

To develop the WS base model, we included the following candidate predictors in a multivariable regression model: maternal age in years, BMI in kg/m^2^, socioeconomic status (high vs low), conception method (assisted, by use of medications such as clomiphene or fertilization procedures including intrauterine insemination, in-vitro fertilization and intracytoplasmic sperm injection, vs natural conception), smoking status (current smokers vs non-smokers), multiple pregnancy (yes vs no); and family history of pre-eclampsia (yes vs no), and ethnicity (Australian/New Zealand-born English-speakers vs immigrants and non-English speakers).

To consider how to deal with the factors measured on a continuous scale (maternal age, BMI) in the model, we graphically examined their relationship with logit pre-eclampsia using a cubic splines approach. We assessed each factor and possible interactions between factors such as maternal age and multiple pregnancy by inspecting their effect size and *p*-value. We manually excluded factors that did not contribute to the model. We then developed the WS base model using the final predictors with no further stepwise procedures.

We performed internal validation of the model using the bootstrapping sampling technique to assess potential overfitting of the regression coefficients [24]. The mean c-statistic (corresponds to the area-under-the curve (AUC)) of the bootstrapping models was compared with the WS base model using the following formula: AUC = 0.5*(Dxy + 1), where: Dxy is Somer’s D. A well fitted model will report minimal optimism. We planned to adjust the regression coefficients by the resulting shrinkage factor if required [24].

To externally validate the base model, we applied the model algorithm in the validation sample. As described above, the base model was developed use in women without high-risk factors, thus we excluded women with high-risk factors from this analysis. We calculated the predicted probability of pre-eclampsia for each individual woman by calculating the (log odds (Y)) and the odds ratio (ExpY) for pre-eclampsia and using the following equation: Probability = odds/1 + odds and presented the distribution of predicted probabilities in a histogram. We assessed model discrimination by calculating the AUC and 95% CI. We assessed model calibration in this sample using the Hosmer-Lemeshow goodness-of-fit test, with *p*-value < 0.05 indicating poor calibration [25]. We also calculated the ratio of observed: expected pre-eclampsia events and graphically assessed calibration by plotting observed risks on the y-axis against predicted risks on the x-axis for subgroups of patients categorized by their predicted probabilities (1- < 2%, 2- < 3%, 3- < 4%, 4- < 5%, 5- < 8%, ≥8%) [26].

#### WS full model

To develop the WS full model, we introduced women with NICE-listed high-risk factors (autoimmune disease, chronic hypertension, chronic renal disease, diabetes) into the model development sample. We developed a multivariable regression model in this sample by retaining the WS base model risk score (Y) and adding the four high-risk factors listed above as additional predictors. We manually excluded high-risk factors that were not strongly or statistically significantly associated with pre-eclampsia. We followed the same approach outlined above for the base model to undertake internal and external validation of the full model to assess potential model overfitting.

#### WS final model

After assessment of over-fitting and calibration of the model in the validation sample, we refitted the WS base and full model in the entire study sample to develop the final WS model. First, we fitted the WS base model in women without high-risk factors. We retained the base model risk score and refitted the WS final model in the entire study sample to estimate the ß-coefficients for the high-risk factors and a new intercept. We presented the intercept and beta (log odd ratio) estimates and 95% CI for the intercept and each predictor.

Given the model is intended to be used to provide pre-eclampsia risk estimates to inform clinical decisions, we also assessed model sensitivity (95% CI), specificity (95% CI), positive predictive value (PPV, 95% CI), negative predictive value (NPV, 95% CI), positive likelihood ratio (LR) and negative LR to predict pre-eclampsia at specified cut-points determined by the risk thresholds for classifying high- vs low-risk. For our primary analysis, we used ≥8% as the risk threshold to classify high-risk as recommended by the United States Preventive Services Task Force (USPSTF) for commencing aspirin prophylaxis based on the prevalence of pre-eclampsia in trials demonstrating the effectiveness of aspirin [4] and from a publication recommending a 6–10% risk threshold for informing aspirin decisions [27]. We also examined the final model performance at 2, 3, 4, 5 and 8% risk thresholds. We also reported model sensitivity at 5 and 10% fixed false positive rates (FPRs) to allow comparison with published models identified from our previous systematic review [13]. For these analyses, we classified women as ‘true positive’ if they had a model-predicted risk above the cut-point and developed pre-eclampsia; false positive (predicted risk at/above cut-point and no pre-eclampsia); true negative (predicted risk below cut-point, no pre-eclampsia) or false negative (predicted risk below cut-point and pre-eclampsia).

In a secondary analysis, we assessed the discrimination of the WS final model to predict early-onset pre-eclampsia and preterm pre-eclampsia by estimating the AUC and sensitivity and specificity at ≥8% risk threshold in the entire study sample.

#### Model comparison with NICE approach

We compared the performance of the WS base and final models with the NICE approach by assessing the sensitivity and specificity, PPV, NPV, positive LR and negative LR of the NICE approach to predict pre-eclampsia in women without high-risk factors for comparison with the WS base model; and all women for comparison with the final model. For these comparisons, we assessed model sensitivity by fixing model specificity at the same level as the NICE approach. We report the model risk threshold that corresponds to this specificity level. For both the WS final model and NICE approach, we also calculated the number needed to treat (NNT) and the number needed to screen (NNS) [28] to avoid one pre-eclampsia event under a strategy where women classified as high-risk are recommended aspirin. For each approach, the NNT was calculated by applying a RR reduction of 10% for aspirin reported from the Perinatal Antiplatelet Review of International Studies (PARIS) individual participant data meta-analysis of randomized controlled trials [21] to the ‘baseline’ risk of pre-eclampsia observed for women classified as high-risk. The NNS was calculated by dividing the NNT by the proportion of pregnant women who were classified as high-risk using the approach.

We performed a secondary analysis to assess the performance of the NICE approach for predicting preterm versus term pre-eclampsia (delivery ≥37 weeks’ gestation) and early-onset versus late-onset pre-eclampsia (delivery ≥34 weeks’ gestation) by estimating the OR and 95% CI using multinomial logistic regression and reporting a *p*-value for the Wald Chi Square test for the hypothesis of no difference in approach performance between the pre-eclampsia subgroups (preterm versus term; and early-onset versus late-onset pre-eclampsia).

We used SPSS version 25 and SAS version 9.3 statistical software and R for all analyses. The R rms package was used for model internal validation (bootstrapping). A p-value of < 0.05 was regarded as statistically significant for all analyses.

We created an Excel spreadsheet to present the WS final model as a risk prediction calculator that can be used in the clinic to provide women with an individualised estimate of their probability of pre-eclampsia [29]. We followed the Transparent Reporting of a multivariable prediction model for Individual Prognosis Or Diagnosis (TRIPOD) guidelines to report our methods and findings [26].

## Results

### Participant characteristics

**Fig. 1:**
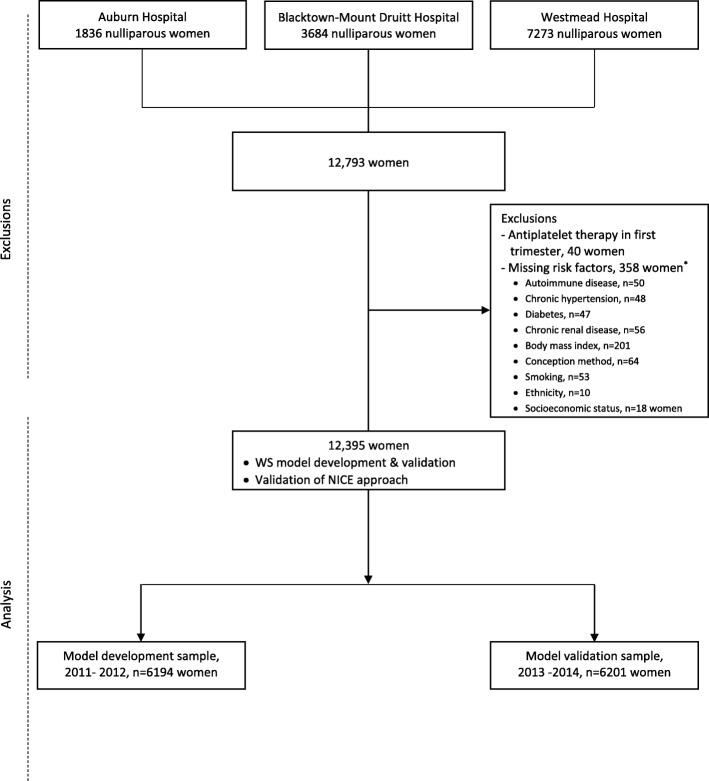
Selection of the study sample for development and validation of the Western Sydney (WS) model and for validation of NICE approach; Western Sydney Local Health District, 2011–2014

A total of 12,793 nulliparous women gave birth in the three hospital sites during the study period. After exclusion of 358 women with missing information on risk factors and 40 women who received aspirin in the first trimester, 12,395 women were included for model development (*n* = 6194) and validation (*n* = 6201) (Fig. 1).

Participant characteristics are summarized in Table 1. Maternal age ranged from 14 to 46 years (mean 27.1, SD 4.9). Overall, 802 (6.5%) women were ≥ 35 years, 1494 (12.1%) were obese (BMI at first visit ≥30 kg/m^2^) and 308 (2.5%) had a multiple pregnancy. Two-thirds of women were immigrants or non-English speakers.

**Table 1:** Characteristics of nulliparous women, WSLHD, 2011-2014. All data are presented as a number (%)

Characteristics	Total (*N* = 12,395)	PE (*N* = 293)	No PE (*N* = 12,102)
Socio-demographic and current pregnancy factors			
*Australian/New Zealand-born English speakers*			
No	8271 (66.7)	141 (48.1)	8130 (67.2)
Yes	4124 (33.3)	152 (51.9)	3972 (32.8)
*Socioeconomic status*^a^			
High	8594 (69.3)	221 (75.4)	8373 (69.2)
Low	3801 (30.7)	72 (24.6)	3729 (30.8)
*Conception method*			
Natural	11,684 (94.3)	259 (88.4)	11,425 (94.4)
Assisted^b^	711 (5.7)	34 (11.6)	677 (5.6)
*Maternal age (years)*			
≤ 24	3678 (29.7)	86 (29.4)	3592 (29.7)
25–29	5067 (40.9)	85 (29.0)	4982 (41.2)
30–34	2848 (23.0)	87 (29.7)	2761 (22.8)
≥ 35	802 (6.5)	35 (11.9)	767 (6.3)
*Body mass index (kg/m*^*2*^*)*			
≤ 24	8255 (66.6)	150 (51.2)	8105 (67.0)
25–29	2646 (21.3)	58 (19.8)	2588 (21.4)
30–34	960 (7.7)	37 (12.6)	923 (7.6)
≥ 35	534 (4.3)	48 (16.4)	486 (4.0)
*Smoking status*			
Non-smokers	11,736 (94.7)	276 (94.2)	11,460 (94.7)
Current smokers	659 (5.3)	17 (5.8)	642 (5.3)
Medical history			
*Autoimmune disease*^c^			
No	12,385 (99.9)	292 (99.7)	12,093 (99.9)
Yes	10 (0.1)	1 (0.3)	9 (0.1)
*Chronic hypertension*			
No	12,296 (99.2)	274 (93.5)	12,022 (99.3)
Yes	99 (0.8)	19 (6.5)	80 (0.7)
*Chronic renal disease*			
No	12,286 (99.1)	279 (95.2)	12,007 (99.2)
Yes	109 (0.9)	14 (4.8)	95 (0.8)
*Diabetes mellitus (type 1 or 2)*			
No	12,326 (99.4)	289 (98.6)	12,037 (99.5)
Yes	69 (0.6)	4 (1.4)	65 (0.5)
*Multiple pregnancy*			
No	12,087 (97.5)	265 (90.4)	11,822 (97.7)
Yes	308 (2.5)	28 (9.6)	280 (2.3)
Family history			
*Family history of PE*			
No	12,361 (99.7)	289 (98.6)	12,072 (99.8)
Yes	34 (0.3)	4 (1.4)	30 (0.2)

^a^Australian Bureau of Statistics Socio-Economic Index for Australia (SEIFA) advantage/disadvantage by postcode classification. Low socioeconomic status = SEIFA scores 1-2; high socioeconomic status = SEIFA scores 3-5.

^b^Assisted by use of medications or fertilization procedures (includes intrauterine insemination, in-vitro fertilization and intracytoplasmic sperm injection).

^c^Autoimmune disease includes systemic lupus erythematosus and antiphospholipid syndrome.

*PE* Pre-eclampsia

Pre-eclampsia incidence was 2.4% (*n* = 293). Forty-six (0.4%) women had early-onset pre-eclampsia and 115 (0.9%) women had preterm pre-eclampsia.

### WS base model

After exclusion of 133 women with high-risk factors, the WS base model was developed in 6061 women. Age was modelled as a continuous spline linear from 27 years; and BMI was modelled as a continuous spline linear from 26.3 kg/m^2^ (Additional file 1: Figure S1). After inspection of effect size and *p*-value for each factor, three factors (conception method, smoking and socioeconomic status) were manually removed. The five factors included in the base model were: maternal age, BMI, ethnicity, multiple pregnancy, and family history of pre-eclampsia (Additional file 2: Table S1).

Internal validation by bootstrapping sampling indicated very small optimism (Dxy 0.009) (Additional file 2: Table S2). The optimism corrected performance estimate of Dxy was 0.3149, which corresponds to an AUC of 0.66, which was similar to the apparent model performance (AUC 0.66).

**Fig. 2:**
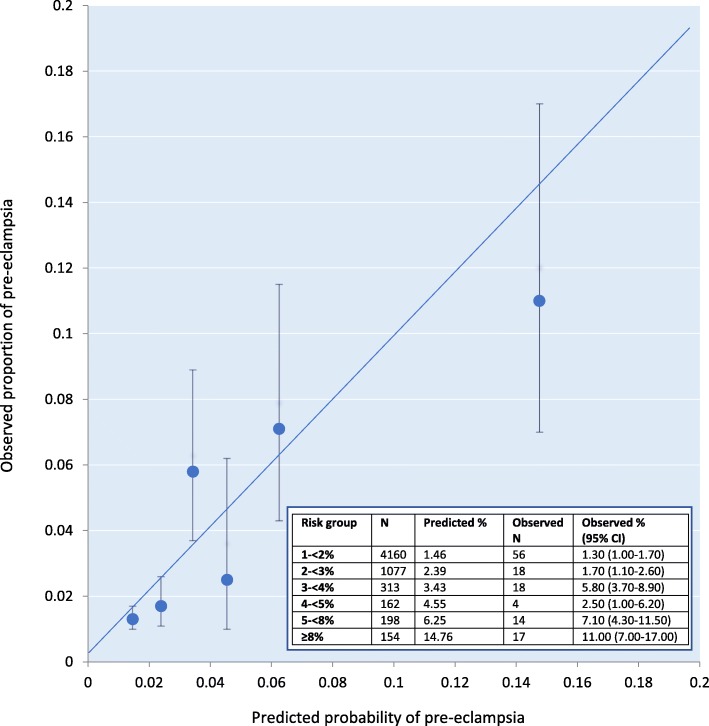
Calibration plot for WS base model in the validation sample; *N* = 6064

After exclusion of 137 women with high-risk factors, 6064 women were included in the external validation sample for the base model. In this sample, the AUC was 0.68 (0.62–0.73) indicating modest discrimination. Base model predictions in the validation sample ranged between 1 and 49% (Interquartile range (IQR): 1.2–2.2%, Additional file 1: Figure S2). Model calibration was good (Hosmer and Lemeshow goodness-of-fit test X^2^ = 6.87; *p* = 0.44); observed:expected ratio of pre-eclampsia events = 0.91. The calibration plot showed an acceptable level of calibration, however, at predicted probabilities for pre-eclampsia higher than 8%, it overestimated the risk of pre-eclampsia (Fig. 2).

### WS full model

The full model was built in the entire model development sample (*n* = 6194). Of the four high-risk factors considered, diabetes (type 1 or 2) was eliminated from the model. The eight factors included in the full model were: maternal age, BMI, ethnicity, multiple pregnancy, and family history of pre-eclampsia, autoimmune disease, chronic hypertension and chronic renal disease (Additional file 2: Table S1).

Internal validation of the full model by bootstrapping sampling indicated very small optimism (Dxy 0.0087) (Additional file 2: Table S2). The optimism corrected performance estimate of Dxy was 3.716000e-01, which corresponds to an AUC of 0.69 which is similar to the apparent model performance (AUC 0.69).

**Fig. 3:**
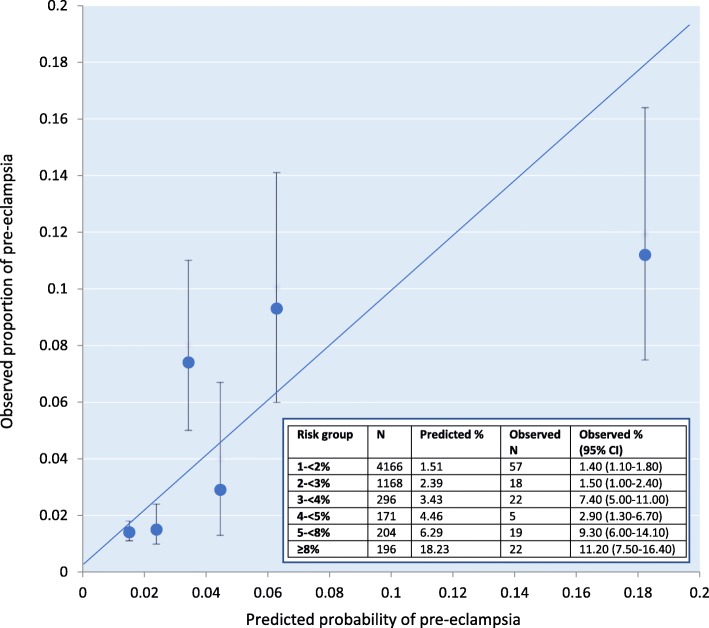
Calibration plot for WS full model in the validation sample; *N* = 6201

In the external validation sample of 6201 women, the AUC was 0.70 (0.65–0.75) indicating good discrimination. Full model predictions in the validation sample ranged between 1 and 86% (IQR: 1.3–2.3%, Additional file 1: Figure S3). Model calibration was good (Hosmer and Lemeshow goodness-of-fit test X^2^ = 9.90; *p* = 0.20); observed:expected ratio of pre-eclampsia events = 0.91. The calibration plot showed an acceptable level of calibration, although as observed for the base model, at predicted probabilities for pre-eclampsia higher than 8%, it overestimated the risk of pre-eclampsia (Fig. 3).

### WS final model

Given the model did not demonstrate over-fitting and was adequately calibrated in the validation sample, the model was refitted in the entire study sample to produce the WS final model.

When applied to the entire study sample, the AUC was 0.70 (0.66–0.73) indicating good discrimination (Additional file 1: Figure S4). The WS final model classified 374 (3%) of women at ≥8% risk of pre-eclampsia (Table 2). At this risk threshold, 54 (14.4%) women develop pre-eclampsia with model sensitivity 18% (14–23%), specificity 97% (97–98%), PPV 14% (11–18%), and NPV 98.0% (97.8–98.3%) The NNT was 69 and NNS was 2295. The performance of the model at lower risk thresholds is shown in Table 2. At fixed 5% FPR, corresponding to a 5.3% risk threshold, the model classified 6% of women to be at high-risk of pre-eclampsia and the sensitivity was 30% (25–36). At a fixed 10% FPR, corresponding to a 3.5% risk threshold, the model classified 11% of women to be at high-risk of pre-eclampsia and the sensitivity was 40% (95% CI 35–46).

**Table 2:** Performance of the WS final model at different risk thresholds, entire study sample (*N* = 12,395)

Risk threshold	PE/n		Sensitivity (95% CI)	Specificity (95% CI)	PPV (95% CI)	NPV (95% CI)	Positive LR (95% CI)	Negative LR (95% CI)
	≥threshold	<threshold						
2%	196/5180	97/7215	67% (61–72%)	59% (58–60%)	4% (3–4%)	99.0% (98.0–99.0%)	1.62 (1.49–1.77)	0.56 (0.48–0.66)
3%	138/1731	155/10664	47% (41–53%)	87% (86–87%)	8% (7–9%)	99.0% (98.0–99.0%)	3.58 (3.14–4.07)	0.61 (0.55–0.68)
4%	105/1098	188/11297	36% (31–41%)	92% (91–92%)	10% (8–11%)	98.0% (98.0–99.0%)	4.37 (3.71–5.15)	0.70 (0.64–0.76)
5%	92/776	201/11619	31% (26–37%)	94% (94–95%)	12% (10–14%)	98.3% (98.0–98.5%)	5.56 (4.62–6.68)	0.73 (0.67–0.79)
8%	54/374	239/12021	18% (14–23%)	97% (97–98%)	14% (11–18%)	98.0% (97.8–98.3%)	6.97 (5.35–9.08)	0.84 (0.79–0.89)

*CI* Confidence interval, *LR* Likelihood ratio, *NPV* Negative predictive value, *PE* Pre-eclampsia, *PPV* Positive predictive value, *WS* Western Sydney

The WS final model can be used to calculate a woman’s probability of developing pre-eclampsia as follows:

Y = − 7.786 + 0.052 * maternal age in years from age 27 years + 0.078 * BMI in kg/m^**2**^ **from 26.3 kg/m**^**2**^ **+ 0.525 if Australian/New Zealand born English-speaker + 1.318 if multiple pregnancy + 1.740 if family history of pre-eclampsia + 1.512 if autoimmune disease + 1.545 if chronic hypertension + 1.494 if chronic renal disease.**

**Odds = Exp** ^**Y (final prediction score)**^

**Pre-eclampsia probability = Odds / (1 + Odds)**

**Fig. 4:**
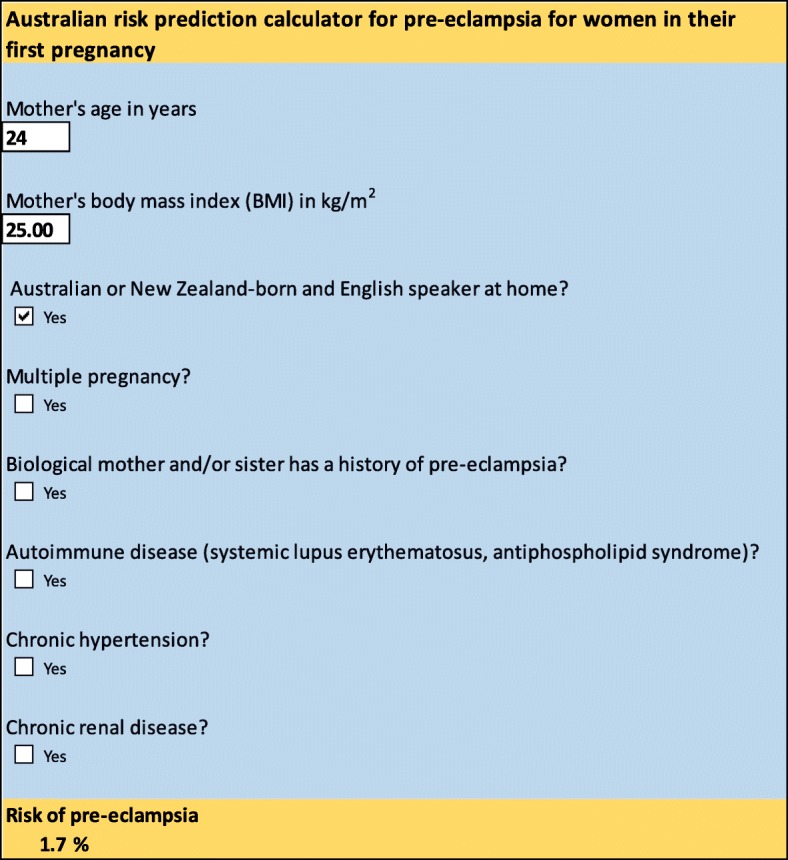
Risk prediction calculator for pre-eclampsia for Australian nulliparous women, as shown in Excel spreadsheet

A ‘WS pre-eclampsia risk prediction tool’ has been created as an Excel spreadsheet that can be used in the clinic to perform these calculations automatically using information entered about a woman’s risk factors (Additional file 3). To illustrate, if a 24 year-old Australian nulliparous woman presents for her first antenatal visit with a BMI of 25 kg/m^2^ and no other risk factor, inputting this value into the Excel spreadsheet gives an estimate of the probability of pre-eclampsia of 1.7% (as shown in Fig. 4). If the same woman also has a family history of pre-eclampsia, addition of this information into the tool gives a revised probability of pre-eclampsia of 8.9%.

### WS model prediction of early-onset and preterm pre-eclampsia

For prediction of early-onset pre-eclampsia, the WS final model had an AUC of 0.72 (95% CI 0.63–0.81). At the ≥8% risk threshold, 17 (4.5%) women developed pre-eclampsia with a model sensitivity of 37% (25–51%) and specificity 97.1% (96.8–97.4%) (Additional file 2: Table S3). At a fixed 5% FPR, corresponding to a 5.6% risk threshold, model sensitivity was 43% (95% CI 30–58). At a fixed 10% FPR, corresponding to a 3.7% risk threshold, model sensitivity was 48% (95% CI 34–62).

For prediction of preterm pre-eclampsia, the WS final model had an AUC of 0.74 (95% CI 0.68–0.79). At the ≥8% risk threshold, 36 (9.6%) women developed pre-eclampsia with a model sensitivity of 31% (24–40%) and specificity 97% (97–98%) (Additional file 2: Table S3). At a fixed 5% FPR, model sensitivity was 44% (95% CI 36–53). At a fixed 10% FPR, model sensitivity was 50% (95% CI 41–59).

### The NICE approach

Using the NICE approach in the subset of women without NICE-listed high-risk factors (*n* = 12,125, pre-eclampsia events = 260), 903 (7.4%) of women were classified screen-positive with a sensitivity of 28% (23–34%) and specificity 93% (93–94%) (Table 3). Among women classified screen-positive, 74 (8.2%) developed pre-eclampsia. For comparison, at the same specificity, the WS base model predicted 7.5% of women in this sample at ≥3.9% risk and achieved similar sensitivity (29%; 24–35%) to the NICE approach. The NNT for women classified by the model at the ≥3.9% risk threshold was 120 and for NICE was 122. The NNS for the model was 1595, compared to 1639 for women using the NICE approach.

**Table 3:** Comparison of the NICE approach versus the WS model for predicting pre-eclampsia in nulliparous women

Approach	Threshold	≥Threshold n (%)	Sensitivity (95% CI)	Specificity (95% CI)	PPV (95% CI)	NPV (95% CI)	Positive LR (95% CI)	Negative LR (95% CI)	NNT	NNS
*Women without high-risk factors, n = 12,125*										
NICE approach	Screen-positive^a^	903 (7.4)	28% (23–34%)	93% (93–94%)	8% (7–10%)	98% (98–99%)	4.07 (3.32–4.99)	0.77 (0.71–0.83)	122	1639
WS base model	3.9%^b^	912 (7.5)	29% (24–35%)	93% (92–93%)	8% (7–10%)	98% (98–99%)	4.15 (3.40–5.07)	0.76 (0.70–0.82)	120	1595
*All women, n = 12,395*										
NICE approach	Screen-positive^a^	1173 (9.5)	37% (31–42%)	91% (91–92%)	9% (8–11%)	98% (98–99%)	4.15 (3.53–4.87)	0.70 (0.64–0.76)	110	1158
WS final model	3.8%^b^	1205 (9.7)	38% (33–44%)	91% (90–91%)	9% (8–11%)	98% (98–99%)	4.23 (3.62–4.95)	0.68 (0.62–0.74)	108	1107

^a^NICE approach screen-positive if ≥1 high-risk factors or ≥ 2 moderate-risk factors. High-risk factors included in this analysis: chronic renal disease, diabetes (type 1 or 2), chronic hypertension and autoimmune disease. Moderate-risk factors included in this analysis: first pregnancy, age ≥ 40 year, body mass index ≥35 kg/m^2^, family history of pre-eclampsia and multiple pregnancy

^b^Model risk cut-off when the model specificity is fixed at the level of the NICE approach

*CI* Confidence interval, *LR* Likelihood ratio, *NICE* National Institute for Health and Care Excellence, *NNS* Number needed to screen, *NNT* Number needed to treat, *NPV* Negative predictive value, *PPV* Positive predictive value, *WS* Western Sydney

Using the NICE approach in the entire study sample including women with high-risk factors (n = 12,395, pre-eclampsia events = 293), 1173 (9.5%) of women were classified screen-positive with a sensitivity of 37% (31–42%), specificity 91% (91–92%) (Table 3). Among women classified screen-positive, 107 (9.1%) developed pre-eclampsia. For comparison, at the same specificity, the WS final model predicted 9.7% of women in this sample at ≥3.8% risk, and achieved a similar sensitivity (38%; 33–44%) to the NICE approach. The NNT for women classified by the model at the ≥3.8 risk threshold was 108 and for NICE was 110. The NNS for the model was 1107, compared to 1158 using the NICE approach.

The accuracy of the NICE approach was higher to predict early-onset and preterm pre-eclampsia (Additional file 2: Table S4).

## Discussion

### Main findings

The WS risk model combines eight routinely collected maternal characteristics to estimate the probability of pre-eclampsia in early pregnancy for women attending antenatal care for their first pregnancy. The model demonstrated good calibration for risk prediction overall, but model accuracy was modest when using the pre-specified 8% risk threshold to predict which women will develop pre-eclampsia. While very few women who did not develop pre-eclampsia had a model-predicted risk above 8% (specificity 97%, 97–98%), only one in five women who developed pre-eclampsia had a risk estimate above this level (sensitivity 18, 95% CI 14–23%). Model sensitivity was higher when assigning a lower risk threshold to classify women as high-risk. For example, one in three women who developed pre-eclampsia had a predicted risk ≥4% (sensitivity 36% (31–41%), specificity 92% (91–92%)). The observed risk of pre-eclampsia was 10% in this group of women.

In contrast to the WS model algorithm, the NICE approach classifies all nulliparous women as moderate-risk, and those with any of the eight additional moderate or high-risk factor/s as high-risk. Based on our study sample, nearly 10% of nulliparous women screened ‘positive’ using the NICE approach, with a sensitivity and specificity for prediction of pre-eclampsia similar to the WS model when at a 3.8% risk threshold (NICE sensitivity 37% (95% CI 31–42%), specificity 91% (91–92%)).

The sensitivity of the WS model was higher for prediction of early-onset and preterm pre-eclampsia than any-onset pre-eclampsia with similar specificity. While these findings might suggest some maternal risk factors are more strongly associated with early-onset and pre-term pre-eclampsia than late-onset pre-eclampsia, an alternative explanation may be that women with risk factors for pre-eclampsia may be more likely to have an induced birth before 40 weeks, thus removing the risk of pre-eclampsia at term. Our finding that the median gestational age at birth for women classified as high-risk (≥1 high-risk factors listed by NICE guideline) was 38.6 weeks’ gestation compared to 39.5 weeks’ gestation for those not classified as high-risk provides some support for this explanation.

The model includes all NICE-listed risk factors except diabetes (type 1 or 2) which did not contribute to the model with an adjusted odds ratio of 0.71 (95% CI 0.22–2.31). One possible interpretation of these findings is that women with well managed diabetes are not at higher risk of pre-eclampsia. Evidence from a clinical trial of women with type 1 diabetes reported each 1% decrement in HbA1C value before pregnancy and at the first antenatal visit was associated with a lower risk of pre-eclampsia supports this as a possible explanation [30]. However, few women in our study sample (< 1%) had diabetes recorded and the wide 95% confidence interval does not exclude the possibility of diabetes as a predictor of pre-eclampsia.

### Comparison with existing evidence

Despite the potential clinical benefits of targeting nulliparous women for pre-eclampsia prevention, most validation studies of risk assessment tools do not report performance separately for nulliparous women [7–12]. Two pre-eclampsia risk prediction models based on routinely collected maternal factors identified from our previous systematic review [13] have been assessed in nulliparous women [6, 31]. Both included more risk factors than the WS model and NICE approach. First, North et al. (2011) recruited 3529 singleton nulliparous women from five centres in Australia, New Zealand, Ireland, and the United Kingdom (UK) and developed a model that included 11 clinical predictors and mean arterial pressure [31]. Model calibration was only reported in the development sample. Model accuracy was not assessed at a clinically-defined risk threshold. At the study-defined cut-off of 25% FPR, model sensitivity (61% (54–68%) [31] was higher than our WS model sensitivity (50% (44–56%) at the same cut-off. This study adds to evidence that combining blood pressure measurements to maternal factors improves prediction of pre-eclampsia in early pregnancy [32]. Unfortunately, blood pressure measurements were not available in the ObstetriX database to allow us to assess the impact on WS model performance.

The second study reported by Wright et al. (2015) assessed the accuracy of a model based on nine maternal factors (maternal age, height, interpregnancy interval, method of conception, chronic hypertension, systemic lupus erythematosus or antiphospholipid syndrome, weight, family history of pre-eclampsia and diabetes mellitus) and ethnicity (white, Afro-Caribbean, South Asian, East Asian and mixed)) [6]. The study included 59,947 singleton nulliparous women recruited from two hospitals in UK. The Wright model was internally validated by 5-fold cross validation. The authors assessed model accuracy at the risk cut-off corresponding to the FPR estimated for the NICE approach and reported a model sensitivity of 31% (28.8–33.3%) at 11.5% FPR which compared favourably to the sensitivity of the NICE approach in the same sample (24.8%, 22.7–26.9%) [6]. The WS final model achieved higher sensitivity to predict pre-eclampsia at the same cut-off (sensitivity 40% (35–46) at 10% FPR).

The Wright model was recently validated in a sample of 4184 nulliparous women recruited from single hospital in the UK [33] to predict preterm pre-eclampsia (preterm pre-eclampsia rate = 0.7%, and compared to the NICE approach. The authors reported higher sensitivity than the original study to predict preterm pre-eclampsia (57.1%; 95% CI 37.5–74.8% vs 35.9; 31.5–40%), with slightly lower FPR (8.8% vs 11.5%), and similar performance to the NICE approach [33]. In comparison, the WS final model showed slightly lower sensitivity to predict preterm pre-eclampsia with narrower confidence interval than the validation study of the Wright model (50%; 41–59% vs 57.1%; 37.5–74.8%) at 8.8% FPR. The accuracy of the NICE approach to predict preterm pre-eclampsia was similar to our present study in Australian women.

One other Australian study has assessed the NICE approach, although the authors did not report results separately for nulliparous women and the sample size of 543 women did not allow precise estimates of performance [12]. We have previously assessed the NICE approach in nulliparous women enrolled in international trials of aspirin prophylaxis who had a relatively high-risk of pre-eclampsia (4.8%) [5]. In this trial sample, the NICE approach had a lower sensitivity (8.9, 10.1%, respectively) and higher specificity (97.2, 96.6%, respectively) than the present study [5]. However, trial data were not available for BMI and not adequately reported for family history which may have resulted in an underestimation of the NICE approach performance [5]. Our present study findings highlight the importance of BMI as a risk factor with obesity (BMI ≥30 kg/m^2^) recorded for 12% women; and we observed a high rate of pre-eclampsia (7%) for this group.

Two international studies have reported on the performance of the NICE approach in nulliparous women, although both excluded women with multiple pregnancies [6, 8]. Both studies reported slightly lower estimates of sensitivity and specificity than the present study. First, a UK study reported the NICE approach classified 12% of nulliparous women as high-risk (similar to 10% in the present study) with a sensitivity of 25% (95% CI 23–27%) and specificity (88%) for prediction of any-onset pre-eclampsia (overall pre-eclampsia rate 2.8%) [6]. Second, a study with smaller sample that included women from the UK, Ireland, New Zealand and Australia reported a sensitivity of 31% and a specificity 88% (calculated from data tabulated) for prediction of preterm pre-eclampsia (overall pre-eclampsia rate 1.3%) [8]. Together with our findings, these results provide consistent evidence that the NICE approach correctly classifies approximately one-quarter to one-third of women who will develop pre-eclampsia as high-risk.

### Limitations

The main limitation of our study is that we collected study data retrospectively which might lead to under-ascertainment of study variables [34]. Under-ascertainment of risk factors may result in underestimation of their predictive performance. Another limitation is that we did not include births from private hospitals to assess whether hospital setting, or maternal factors related to delivery in a private hospital, impacts the risk of developing pre-eclampsia which would reduce the applicability of the WS model to women giving birth outside public hospitals. Finally, no women in the study sample had more than three high-risk factors recorded, thus model predictions cannot be extended to such women.

### Clinical implications

The main intended benefit of pre-eclampsia risk assessment is to correctly identify high-risk women who will benefit from prophylaxis and/or further testing and management by a specialist to prevent the condition or its complications. Although we demonstrate the predictive performance of the WS model and NICE approach is modest compared to that reported for strategies using tests such as uterine artery Doppler and serum biomarkers [13], our findings support the clinical value of these approaches in settings where such specialised tests are not routinely available. For example, the NICE approach can be recommended for nulliparous women, if one considers managing eleven women as high-risk for every one woman who will develop pre-eclampsia is acceptable (PPV 9% (8–11%)). Acceptability will depend on the level of concern about the potential adverse effects of prophylactic agents, such as aspirin [35]. In settings where specialised tests are available, the NICE approach may have a role to select high-risk women for further testing. Under this strategy, our estimate of NPV at 98% indicates that for every 50 women classified as low-risk using the NICE approach, one woman will develop pre-eclampsia and miss the opportunity for further testing.

Compared to the NICE approach, the WS model has the advantage of providing an estimate of the probability of pre-eclampsia to assist patient counselling and guide clinical practice decisions about prophylaxis and/or referral for further testing. In particular, the model allows risk prediction based on maternal age and BMI, which are more common than high-risk factors such as chronic hypertension, whereas the NICE approach is restricted to using a single different cut-off for these two factors. We provide the model algorithm in an Excel spreadsheet that can be readily incorporated into the first antenatal clinic visit. At a population level, setting the risk threshold of 4% to guide decisions such as aspirin prophylaxis appeared appropriate with a 10% observed risk of pre-eclampsia in this group of women.

### Research implications

The WS model should be validated outside of the WSLHD before implementing for nulliparous women in other settings. For example, our findings that immigrant women are at lower risk of pre-eclampsia may not apply outside of Australia.

Further research is needed to investigate whether the inclusion of individual ethnic groups may improve model prediction in Australia’s highly culturally diverse antenatal population, and to investigate the value of including maternal blood pressure measurement in the model.

In settings such as Australia, where tests such as uterine artery Doppler and serum biomarkers are available for pre-eclampsia risk assessment but not yet widely used, broader implementation will depend on cost-effectiveness relative to risk assessment without these tests. Our findings of the accuracy of simple risk tools will be valuable to inform these cost-effectiveness analyses. The WS model can also be used as a foundation on which to build a specialised risk prediction model.

## Conclusions

A risk prediction model that incorporates guideline-listed risk factors and ethnicity provides modest performance for predicting pre-eclampsia in Australian nulliparous women. The model did not outperform the NICE approach, but has the advantage of providing individualised risk estimates over the NICE guideline to assist patient counselling and inform decisions for further testing and prophylaxis.

## Supplementary information

**Additional file 1: Figure S1.** Fitted trend between a continuous predictor and the logit pre-eclampsia using cubic spline function, *Legend:* (A) maternal age in years; (B) body mass index in kg/m^2^; all women (*N* = 12,395). **Figure S2.** Distribution of predicted probabilities for pre-eclampsia using the WS base model in the validation sample excluding women with high-risk factors (*N* = 6064). **Figure S3.** Distribution of predicted probabilities for pre-eclampsia using the WS full model in the validation sample (*N* = 6201). **Figure S4** Receiver-operator characteristic curve for the WS final model for prediction of pre-eclampsia in the entire study sample (N = 12,395), area-under-curve 0.70 (95% CI 0.66–0.73).

**Additional file 2: Table S1.** Western Sydney (WS) model for prediction of pre-eclampsia in women without known high-risk factors, and all women, model development sample. **Table S2.** Internal validation of Western Sydney (WS) base and full model by bootstrapping, 1000 resamples. **Table S3.** Performance of the Western Sydney (WS) final model for prediction of early-onset pre-eclampsia and preterm pre-eclampsia at ≥8% risk threshold, *N* = 12,395. **Table S4.** Performance of NICE approach* to predict preterm, term, early- and late-onset pre-eclampsia for nulliparous women in WSLHD, 2011–2014 (*n* = 12,395).

**Additional file 3.** Australian risk prediction calculator for pre-eclampsia for women in their first pregnancy.

## Data Availability

The data and analyses that support the findings of this study are available on request from the co-author SL; email: sally.lord@nd.edu.au. The data are not publicly available due to WSLHD regulations.

## Abbreviations

aOR: Adjusted odds ratio
AUC: Area under the curve
BMI: Body mass index
CI: Confidence interval
FPR: False positive rate
IQR: Interquartile range
LR: Likelihood ratio
NICE: National Institute for Health and Clinical Excellence
NNS: Number needed to screen
NNT: Number needed to treat
NPV: Negative predictive value
PARIS: Perinatal Antiplatelet Review of International Studies
PE: Pre-eclampsia
PPV: Positive predictive value
ROC: Receiver operating characteristics
SD: Standard deviation
SEIFA: Socio-economic Advantage and Disadvantage from the Socio-Economic Indexes for Areas
SOMANZ: Society of Obstetric Medicine of Australia and New Zealand
TRIPOD: Transparent Reporting of a multivariable prediction model for Individual Prognosis Or Diagnosis
USPSTF: United States Preventive Services Taskforce
WS: Western Sydney
WSLHD: Western Sydney Local Health District
