# Supplementary material for: Prediction of pre-eclampsia in nulliparous women using routinely collected maternal characteristics: a model development and validation study
Source: BMC Pregnancy Childbirth. 2020 Jan 6;20:23. doi: 10.1186/s12884-019-2712-x (PMC6945640; doi:10.1186/s12884-019-2712-x)
Supplement: Supplementary file 1 — Additional file 1: Figure S1. Fitted trend between a continuous predictor and the logit pre-eclampsia using cubic spline function, Legend: (A) maternal age in years; (B) body mass index in kg/m2; all women (N = 12,395). Figure S2. Distribution of predicted probabilities for pre-eclampsia using the WS base model in the validation sample excluding women with high-risk factors (N = 6064). Figure S3. Distribution of predicted probabilities for pre-eclampsia using the WS full model in the validation sample (N = 6201). Figure S4 Receiver-operator characteristic curve for the WS final model for prediction of pre-eclampsia in the entire study sample (N = 12,395), area-under-curve 0.70 (95% CI 0.66–0.73). [file 12884_2019_2712_MOESM1_ESM.docx]

**A.**


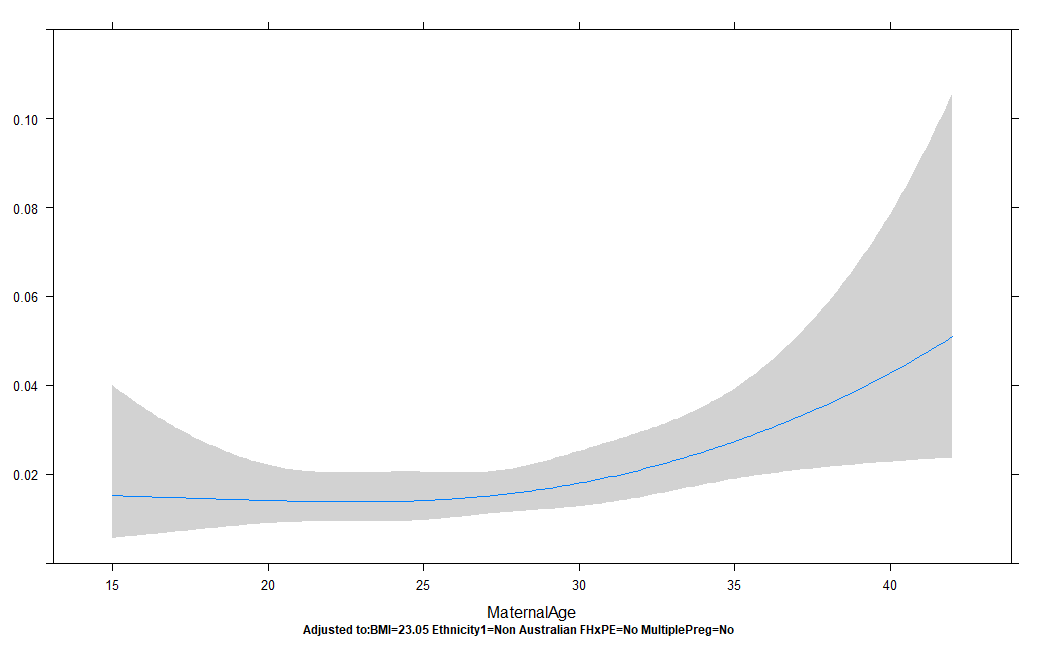


Probability of pre-eclampsia

Maternal age (years)

**B.**


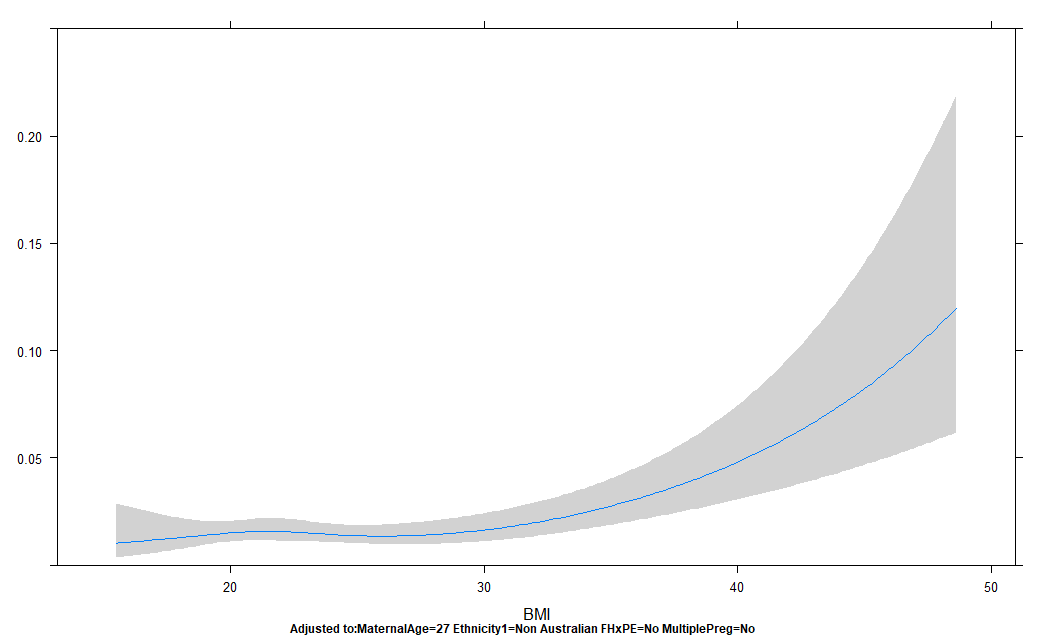


Probability of pre-eclampsia

Body Mass Index (kg/m^2^)

**Fig. S1** Fitted trend between a continuous predictor and the logit pre-eclampsia using cubic spline function, ***Legend:*** (A) maternal age in years; (B) body mass index in kg/m^2^; all women (N=12,395)


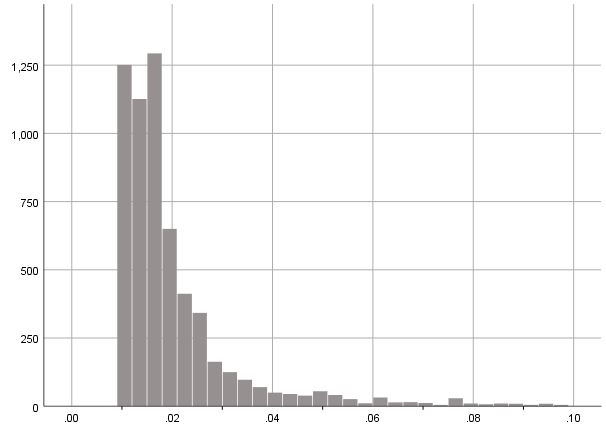


Frequency of women

Predicted probability of pre-eclampsia

**Fig. S2** Distribution of predicted probabilities for pre-eclampsia using the Western Sydney (WS) base model in the validation sample excluding women with high-risk factors (N=6064)


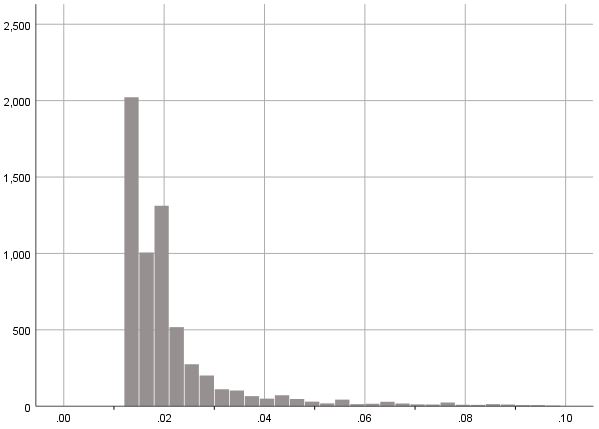


Frequency of women

Predicted probability of pre-eclampsia

**Fig. S3** Distribution of predicted probabilities for pre-eclampsia using the Western Sydney (WS) full model in the validation sample (N=6201)


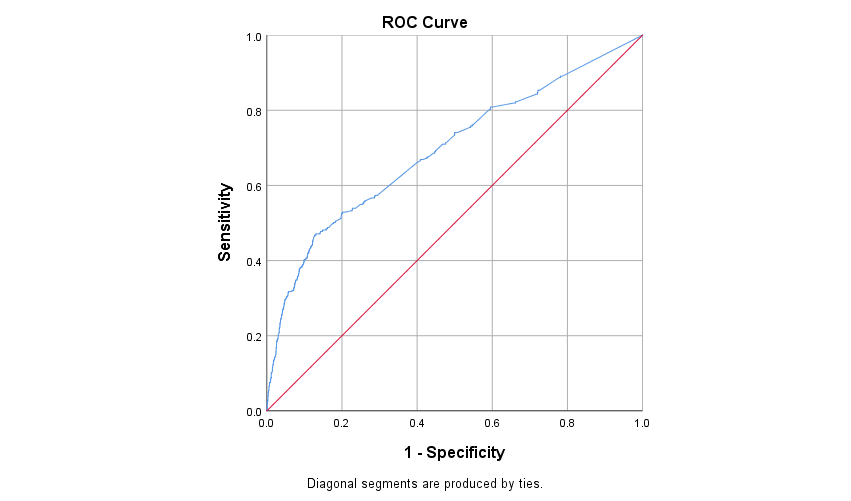


**Fig. S4** Receiver-operator characteristic curve for the Western Sydney (WS) final model for prediction of pre-eclampsia in the entire study sample (N=12,395), area-under-curve 0.70 (95% CI 0.66-0.73)
