# Supplementary material for: Prediction of pre-eclampsia in nulliparous women using routinely collected maternal characteristics: a model development and validation study
Source: BMC Pregnancy Childbirth. 2020 Jan 6;20:23. doi: 10.1186/s12884-019-2712-x (PMC6945640; doi:10.1186/s12884-019-2712-x)
Supplement: Supplementary file 2 — Additional file 2: Table S1. Western Sydney (WS) model for prediction of pre-eclampsia in women without known high-risk factors, and all women, model development sample. Table S2. Internal validation of Western Sydney (WS) base and full model by bootstrapping, 1000 resamples. Table S3. Performance of the Western Sydney (WS) final model for prediction of early-onset pre-eclampsia and preterm pre-eclampsia at ≥8% risk threshold, N = 12,395. Table S4. Performance of NICE approach* to predict preterm, term, early- and late-onset pre-eclampsia for nulliparous women in WSLHD, 2011–2014 (n = 12,395). [file 12884_2019_2712_MOESM2_ESM.docx]

**Table S1** Western Sydney (WS) model for prediction of pre-eclampsia in women without known high-risk factors, and all women, model development sample

| **Predictors** | **B** | **SE** | **aOR (95% CI)** | **P-value** |
| --- | --- | --- | --- | --- |
| ***WS base model developed in women without high-risk factors (N=6061)*** | | | | |
| **Intercept** | -8.963 | 0.765 |  | <0.001 |
| **Maternal age*** | 0.079 | 0.023 | 1.08 (1.03-1.13) | 0.001 |
| **BMI**** | 0.093 | 0.017 | 1.10 (1.06-1.13) | <0.001 |
| **Australian/NZ ethnicity** | 0.452 | 0.188 | 1.57 (1.09-2.27) | 0.02 |
| **Multiple pregnancy** | 1.499 | 0.308 | 4.48 (2.45-8.18) | <0.001 |
| **Family history of PE** | 2.553 | 0.658 | 12.85 (3.54-46.64) | <0.001 |
|  |  |  |  |  |
| ***WS full model developed in all women (N=6194)*** | | | | |
| **Intercept** | -0.444 | 0.346 |  | 0.20 |
| **WSO base model risk score** | 0.885 | 0.094 | 2.42 (2.02-2.91) | <0.001 |
| **Autoimmune disease** | 2.010 | 1.169 | 7.46 (0.75-73.86) | 0.086 |
| **Chronic hypertension** | 1.899 | 0.410 | 6.68 (2.99-14.91) | <0.001 |
| **Chronic renal disease** | 1.504 | 0.425 | 4.50 (1.96-10.35) | <0.001 |

*Continuous spline linear from 27 years.

**Continuous spline linear from 26.3 kg/m^2^.

aOR = Adjusted odds ratio, BMI = Body mass index, CI = Confidence interval, NZ = New Zealand, PE = Pre-eclampsia, SE = Standard error

**Table S2** Internal validation of Western Sydney (WS) base and full model by bootstrapping, 1000 resamples

| **index.orig training test optimism index.corrected n** |
| --- |
| ***WS base model in women without high-risk factors (N=6061)**** |
| **Dxy**  0.3239 0.3247 0.3157 0.0090 0.3149 913 |
| **R2** 0.0720 0.0750 0.0671 0.0079 0.0641 913 |
| **Intercept**  0.0000 0.0000 -0.1066 0.1066 -0.1066 913 |
| **Slope** 1.0000 1.0000 0.9687 0.0313 0.9687 913 |
| **B** 0.0208 0.0208 0.0209 -0.0001 0.0210 913 |
|  |
| ***WS full model in all women (N=6194)***** |
| **Dxy**  0.3803 0.3820 0.3733 0.0087 3.716000e-01 893 |
| **R2**  0.0910 0.0935 0.0848 0.0087 8.230000e-02 893 |
| **Intercept**  0.0000 0.0000 -0.1283 0.1283 -1.283000e-01 893 |
| **Slope**  1.0000 1.0000 0.9622 0.0378 9.622000e-01 893 |
| **B**  0.0228 0.0228 0.0229 -0.0002 2.300000e-02 893 |
|  |

*Divergence or singularity in 87 samples.

** Divergence or singularity in 107 samples.

**Table S3** Performance of the Western Sydney (WS) final model for prediction of early-onset pre-eclampsia and preterm pre-eclampsia at ≥8% risk threshold, N=12,395

| **Test characteristic** | **n/N** | **Predictive accuracy** |
| --- | --- | --- |
| ***Early-onset pre-eclampsia*** |  |  |
| Sensitivity (95% CI) | 17/46 | 37% (25-51%) |
| Specificity (95% CI) | 11992/12349 | 97.1% (96.8-97.4%) |
| PPV (95% CI) | 17/374 | 5% (3-7%) |
| NPV (95% CI) | 11992/12021 | 99.8% (99.7-99.8%) |
| Positive LR (95% CI) | **-** | 12.78 (8.65-18.90) |
| Negative LR (95% CI) | - | 0.65 (0.52-0.81) |
|  |  |  |
| ***Preterm pre-eclampsia*** |  |  |
| Sensitivity (95% CI) | 36/115 | 31% (24-40%) |
| Specificity (95% CI) | 11942/12280 | 97% (97-98%) |
| PPV (95% CI) | 36/374 | 10% (7-13%) |
| NPV (95% CI) | 11942/12021 | 99.3% (99.2-99.5%) |
| Positive LR (95% CI) | - | 11.37 (8.51-15.21) |
| Negative LR (95% CI) | - | 0.71 (0.62-0.80) |
|  |  |  |

CI = Confidence interval, LR = Likelihood ratio, NPV = Negative predictive value, PPV = positive predictive

**Table S4** Performance of NICE approach* to predict preterm, term, early- and late-onset pre-eclampsia for nulliparous women in WSLHD, 2011-2014 (n=12 395)

| **Risk rule** | **N**** | **PE**  **n** | **OR**  **(95% CI)** | **P-value** | **Sensitivity**  **(95% CI)** | **Specificity**  **(95% CI)** | **PPV**  **(95% CI)** | **NPV**  **(95% CI)** | **Positive LR**  **(95% CI)** | **Negative LR**  **(95% CI)** |
| --- | --- | --- | --- | --- | --- | --- | --- | --- | --- | --- |
| ***Preterm pre-eclampsia requiring delivery before 37 weeks’ gestation*** | | | | | | | | | | |
| negative  positive | 11091  1126 | 55  60 | 1.00  11.29 (7.79–16.37) | <0.001^a^  <0.001^b^ | 52% (43–61%) | 91% (91–92%) | 5% (4–7%) | 99.5% (99.4–99.6%) | 5.92 (4.93–7.12) | 0.52 (0.43–0.64) |
| ***Term pre-eclampsia with delivery at or after 37 weeks’ gestation*** | | | | | | | | | | |
| negative  positive | 11167  1113 | 131  47 | 1.00  3.71 (2.65–5.21) | <0.001 | 26% (21–33%) | 91% (91–92%) | 4% (3–6%) | 98.8% (98.6–99.0%) | 3.00 (2.33–3.86) | 0.81 (0.74–0.88) |
| ***Early-onset pre-eclampsia requiring delivery before 34 weeks’ gestation*** | | | | | | | | | | |
| negative  positive | 11060  1088 | 24  22 | 1.00  9.49 (5.30–16.98) | <0.001^a^  0.09^c^ | 48% (34–62%) | 91% (91–92%) | 2% (1–3%) | 99.8% (99.7–99.9%) | 5.43 (3.99–7.38) | 0.57 (0.43–0.76) |
| ***Late-onset pre-eclampsia requiring delivery at or after 34 weeks’ gestation*** | | | | | | | | | | |
| negative  positive | 11198  1151 | 162  85 | 1.00  5.43 (4.15–7.12) | <0.001 | 34% (29–41%) | 91% (91–92%) | 7% (6–9%) | 98.6% (98.3–98.8%) | 3.91 (3.26–4.68) | 0.72 (0.66–0.79) |

*NICE approach: Positive refers to ≥1 high-risk factors or ≥2 moderate-risk factors. High-risk factors included in this analysis: chronic renal disease, diabetes (type 1 or 2), chronic hypertension and autoimmune disease status. Moderate-risk factors included in this analysis: first pregnancy, age ≥40 year, body mass index ≥35 kg/m^2^, family history of pre-eclampsia and multiple pregnancy.

**Excludes women with missing data.

^a^P-value for test of null hypothesis of no difference in odds of pre-eclampsia for guideline approach positive versus negative.

^b^P-value for test of null hypothesis of no difference in odds ratio for guideline positive versus negative between pre-eclampsia with delivery <37 versus ≥37 weeks; Wald Chi square=19.39

^c^P-value for test of null hypothesis of no difference in odds ratio for guideline positive versus negative between pre-eclampsia with delivery <34 versus ≥34 weeks; Wald Chi square=2.96

CI = Confidence interval, LR = Likelihood ratio, NICE = National Institute for Health and Care Excellence, NPV = Negative predictive value, OR = Odds ratio, PE = Pre-eclampsia, PPV = Positive predictive value
